# Continuous Gum Teeth

**Published:** 1855-04

**Authors:** 


### ARTICLE XIII.

### *Continuous Gum Teeth.*

UNTIL very recently we have had much trouble in obtaining teeth that would best suit this kind of work, those in market having been found objectionable, either from the position of the platina pins, or from their shape or color.

We were troubled principally from the pins coming up too high, so as to expose the platina backings above the composition, to obviate which, the gum would have to be placed above the crown of the tooth, making a clumsy and unsightly finish, as well as producing a heavy and unpleasant effect.

Again, the depressions made for the backings were not deep enough, for when a tooth was backed, the platina extended beyond the prominence of the crown, not allowing room to shape the gum, as would appear natural, or feel comfortable to the tongue, but leave a protuberance very objectionable, as it afforded no additional strength.

No attention had been paid to the relative depth of the backing allowed in the cuspid and bicuspid teeth, so that there must almost invariably be left an ugly jog, from the differing length of the backings at this point, the bicuspids being generally one-eighth of an inch longer, instead of the same length, if possible. And here, particularly, the difficulty was found of the depression in the teeth ; for the backing, being made too shallow, in many teeth hardly perceptible, instead of *at least* one-third of the thickness of the tooth.

Having been consulted upon this subject by Messrs. Orum & Armstrong, of Philadelphia, I have received some manufactured by them, greatly freed from these objections.

The shades of color and general form of their teeth are beautiful, and will, no doubt, be readily appreciated by the profession, as they are certainly great improvements upon all previously manufactured, which we have had the oppportunity of using. We, therefore, recommend them cheerfully to our friends and the profession, whilst expressing the hope that these improvements may be persevered in until even a greater degree of perfection has been reached.

A. A. B.

### REVIEW DEPARTMENT.

